# Ecodesign of the Aluminum Bronze Cutting Process

**DOI:** 10.3390/ma15082735

**Published:** 2022-04-08

**Authors:** Dan Dobrotă, Mihaela Oleksik, Anca Lucia Chicea

**Affiliations:** Faculty of Engineering, University Lucian Blaga of Sibiu, Str. Emil Cioran, Nr.4, 550025 Sibiu, Romania; mihaela.oleksik@ulbsibiu.ro (M.O.); anca.chicea@ulbsibiu.ro (A.L.C.)

**Keywords:** ecodesign, aluminum bronze, cutting, force measurement, roughness

## Abstract

The realization of products from materials with high properties generally involves very high energy consumption. Thus, in the research, it was considered to optimize the machining process by cutting of an aluminum bronze alloy, so as to obtain a reduction in energy consumption in correlation with the roughness of the machined surfaces. The research focused on the processing of a semi-finished product with a diameter of Ø = 20 mm made of aluminum bronze (C62300). In addition, in the research, the aim was to establish some correlations between the amount of power consumed and the quality of the surfaces processed by cutting. In this sense, the forces were measured in the 3 directions specific to the cutting process (*F_c_*; *F_f_*; *F_p_*) for 3 tools construction variants and power consumed. The results showed that, if a certain constructive variant of the cutting tool is used in the processing, a reduction of the power consumed to cutting can be obtained by approximately 30% and a reduction of the roughness of the processed surface by approximately 90–100%. Furthermore, following the statistical processing of the results, it was shown that it would be advisable to use, especially in roughing processes, the cutting tool variant that offers the greatest reduction in roughness and cutting power.

## 1. Introduction

The constant increase in energy consumption and its costs has led to a strong increase in the costs of manufacturing products [1]. Thus, over time, various research have presented studies on the energy consumption of machine tools in the processing of various materials by cutting to obtain certain products [2,3]. In this sense, research was carried out which allowed to establish correlations between the amount of energy consumed and the quality and precision of the surfaces processed by cutting. Thus, it has been demonstrated that the lowest possible energy consumption can be obtained in the conditions in which optimal parameters are established for the cutting process, but also an optimal functional geometry of the cutting tool, depending on the quality of the removable plate. In addition, an optimal choice of cutting process parameters and tools can generate energy savings of up to 6–40% [4,5].

For the cutting processing of various materials, a series of studies have been carried out on the possibilities of reducing energy consumption, but there are still a number of materials for which no technical solutions have been found to ensure the best processing conditions. Aluminum alloys fall into the category of materials that are difficult to cut by cutting and which require very high energy consumptions [6]. These alloys are widely used in the machine building industry, namely for the manufacture of: parts for aircraft, components for ships; tools for plastic deformation of sheets, etc. This type of material is characterized by the fact that it has very good mechanical properties and a high resistance to corrosion. The majority of aluminum-bronze alloys have a chemical composition in which aluminum is 5% to 11% by weight, the rest of the composition being represented by copper, but also by other alloying elements such as iron, nickel, manganese and silicon. It also has fairly good machining qualities when using cutting edge tools. Due to the danger of heating the part during cutting, it is recommended to use a coolant. Under these conditions, the machining of the external surfaces of the parts can be carried out at quite high speeds, and for the internal one’s lower speeds are recommended (drilling, reaming), because there is a danger that the bronze chips will adhere to the tool faces [7,8,9].

Given the category of parts for which this material is used, in many cases, it is necessary that the parts be processed by cutting so as to obtain a roughness as low as possible and an accuracy as good as possible [10,11]. Furthermore, as in all processing processes, and in the case of cutting by processing this type of material, it is necessary to obtain the lowest possible energy consumption. The roughness of the surface of the machined parts depends on a multitude of factors, but the most important are: the functional geometry of the tool, the parameters of the cutting process, the type of cooling-lubrication fluid used, etc [12]. 

A major problem with aluminum bronze machining is the adhesion of the machined material to the active surfaces of the tool used in machining. This adhesion has negative effects on both the roughness and the accuracy of the machined surface [13,14]. In this regard, in some research, tools from various high-speed steel (HSS) tool and YW1 cemented carbide materials have been tested and a reduction in adhesions has been observed when using the tool made from YW1, but not a complete elimination of them. Thus, the use of tools made of various materials is not a solution to eliminate adhesions in the conditions in which and other parameters of the cutting process are not optimized [15].

In order to improve the machining conditions of aluminum bronze, in some research, it has been proposed to use a jet of high-pressure coolant that is inserted between splinter and tool. This leads to an improvement in the material’s processing capacity and a considerable reduction in the temperature of the technological system. However, this technological solution involves a series of expensive technological equipment and obtaining a fairly high roughness for the processed surfaces [16,17]. 

Tungsten carbide tools can be used in good condition for machining parts made of C95800 aluminum bronze, and high-speed machining has shown that surfaces with low roughness and relatively good accuracy can be obtained. In addition, wear rates in carbide tools are quite low under optimal cutting regimes [18,19]. 

Furthermore, for the turning processing of materials, with a lowest possible energy consumption, it is necessary to adopt an efficient technique for assessing the characteristics of energy consumption in processing in order to select the technological process of processing with high energy efficiency, taking into account and processing accuracy. There are currently three representative methods for predicting energy consumption during turning: the method based on specific energy (SEM), the method based on the cutting force (CFM), and the method based on exponential function (EFM). SEM considers that the material removal power is the product of the specific cutting energy and the material removal rate (MRR). Improper application of these methods can lead to low prediction accuracy, which cannot support accurate energy assessment and reduction of consumed energy in processing [20,21].

In addition to these methods of evaluating the energy consumption of turning, empirical models for predicting energy consumption can be considered [22,23] and, more recently, neural prediction models with inverse propagation [24,25]. However, even these methods based on neural networks cannot take into account all the parameters that accompany a cutting process and, generally, only consider the parameters of the cutting regime (cutting speed, feed rate, depth of cut) [26]. Optimizing the values of the cutting regime parameters can lead to a reduction in energy consumption, but, if only these parameters are taken into account, a more complex analysis of the energy problems that may occur at cutting can not be performed. For example, the level of wear of the cutting tool must also be taken into account. Thus, its increased wear can lead to a rapid increase in energy consumption. Under these conditions, it is necessary to constantly monitor the wear of the cutting tool, but also the adoption of certain tool construction systems to allow the registration of a wear as low as possible. The development of various construction tools for cutting tools has made it possible to achieve certain results in the machining process, but all these tool systems need to be improved to achieve new industry-specific performance 4.0 [27]. The design of cutting tools to ensure a reduction in energy consumption in correlation with the good quality of the surfaces of the cutting conditions is an important objective of the research of cutting processing [28,29].

In view of the above, it is necessary to find the best conditions for cutting by processing in order to obtain the lowest possible energy consumption and the best possible quality of the processed surfaces. Thus, for the production process itself, it is necessary to implement certain conditions that allow the optimization of energy consumption, considering the complexity of the cutting processes. The research was focused on establishing the conditions of machining by turning aluminum bronze so as to obtain the lowest energy consumption and the best quality of the processed surfaces. 

## 2. Materials and methods

### 2.1. Materials Used in Research

In order to be able to establish the best conditions for processing aluminum bronze, the research considered the processing by turning of cylindrical specimens with diameter Ø = 20 mm made of aluminum bronze (C62300). The choice of aluminum bronze was made considering the fact that this material is one used for the realization of complex parts of special importance in the aeronautical, naval, etc. industry. The material used in the research (C62300) comes from Aviva Metals, Houston, TX, USA. The chemical composition and mechanical properties of aluminum bronze are shown in Table 1.

### 2.2. Equipment and Tools Used in the Processings by Cutting

**Figure 1:**
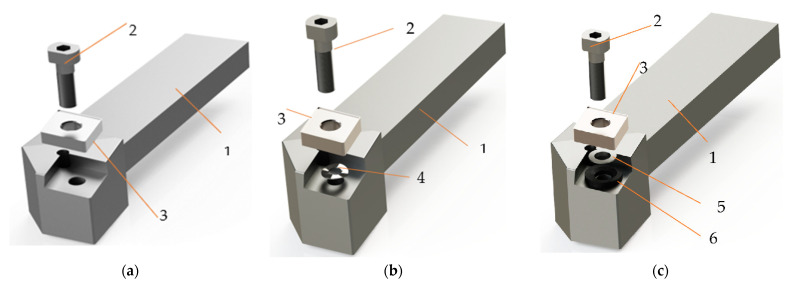
Tools for longitudinal turning: (**a**)—in the classic version (V01); (**b**)—with improved constructive form with a spring washer (V02), (**c**)—with improved constructive form with two spring washers (V03), 1—knife body; 2—screw fixing; 3—removable plate, 4—spring washer, 5—spherical washer, 6—spherical washer holder.

Experimental research was performed on a numerically controlled lathe (CNC). The use of this type of equipment was imposed by the fact that it allows an adjustment in a very large range of the parameters of the cutting process. Thus, the CNC lathe used was one coded VT 15 PLUS with numerical control FANUC, provided by FANUC Automation Romania S.R.L., Cluj, Romania. A longitudinal turning tool was used in the machining (Figure 1). A tool body with a 20 mm × 20 mm section provided by Sandvik-Coromant, Brașov, Romania, was used to obtain the cutting tools used in processing. In addition, the equipment of the cutting tool body was made with a plate SNMG 12 04 12-PMC - Sandvik Coromant with the parameters presented in Table 2.

Aluminum bronze alloys are quite difficult to process by cutting, especially if it is necessary to obtain good qualities of the machined surfaces. The problems that arise in the processing of bronze-aluminum alloys are determined by the mechanical properties of this material that influence the process of chip formation. Thus, during chipping some difficulties may arise in the process of chip formation caused mainly by the phenomenon of deposits on the edge. All this can also lead to an increase in energy consumption due to the increase in the values of the shear forces as a result of the intensification of the friction phenomena. All these phenomena also appear in the longitudinal turning, and for the improvement of the conditions of processing by cutting in the research were used three variants of cutting tools, Figure 1. Thus, the first variant of tool used, Figure 1a; it was the one in which it has the classical constructive form. In order to avoid the phenomenon of deposits on the edge and to improve the conditions of cutting processing, two other cutting tools V02, Figure 1b, V03, Figure 1c were used in the research. These two tool variants were made by placing under the removable plate of a constructive elements, in way to avoid the appearance of deposits on the edge by the adhesion of a part of the material from splinter on the surface of the tool.

**Figure 2:**
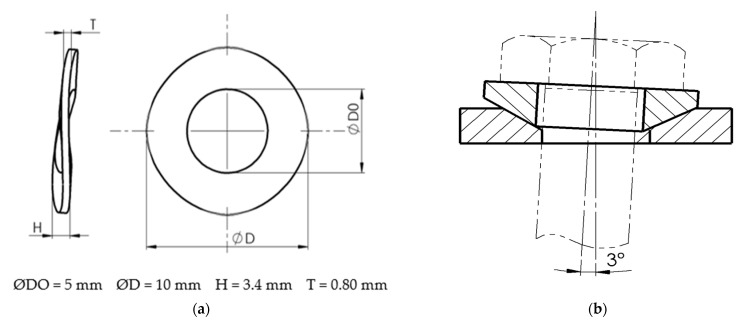
Constructive types of elements used to make tools: (**a**)—corrugated spring washer—for tool V02; (**b**)—spherical washer—conical base—for tool V03.

The control of the deposition phenomenon on the edge allows both a considerable reduction of the frictional forces that appear during the cutting and a considerable improvement of the quality of the processed surfaces. A reduction in friction forces results in a considerable reduction in the total cutting force and, consequently, in the energy consumed in cutting. Thus, during the research, a series of constructive improvements were proposed for the cutting tool. These constructive improvements can ensure an optimal functional geometry for the cutting tool according to the actual cutting conditions. In the case of the V02 cutting tool, a corrugated spring washer was placed under it removable plate, Figure 2a, which allows a continuous change of the tool geometry. When there is a tendency for deposits to appear on the edge, the frictional forces increase, causing a deformation of the spring washer and a change in the tool geometry. In the case of variant V03, a conical base spherical washer was placed under the tool plate, Figure 2b, which allows a change in the position of the plate axis by 3°. This enables the cutting tool to change its geometry during operation and in particular the clearance angle and the seating angle. The corrugated spring washer used corresponds to DIN 137 B, steel A2 1.4305 and the spherical washer with conical base corresponds to DIN 6319, steel 1.4305 and are manufactured by Parcon Freiwald, Germany.

In order to carry out the experimental research, the parameters of the cutting regime were established taking into account the recommendations from the specialized literature [19]. In view of the processing conditions, the following parameters of the cutting regime have been established: rotational speed *n* = 800–1200 rpm, processing depth *a_p_* = 0.4–0.8 mm, longitudinal advance *f* = 0.15–0.25 mm/rot. Since the main objective of the paper is to analyze how the construction of the cutting tool can influence the energy consumed in cutting and the quality of the processed surfaces, the method of factorial experiments for the programming of experiments was used in the research. Thus, for the three parameters (*n*, *a_p_*, *f*) two levels were considered (maximum and minimum), depending on which the cutting force (calculated power at cutting) was observed, respectively, the roughness of the processed surfaces for the three variants of cutting tools. In these conditions, considering the fact that 3 variable parameters were established, each having 2 levels, 8 types of samples were made, according to those presented in Table 3.

### 2.3. Analysis of the Evolution Forțelor Și Puterii la Strunjirea Pieselor

**Figure 3:**
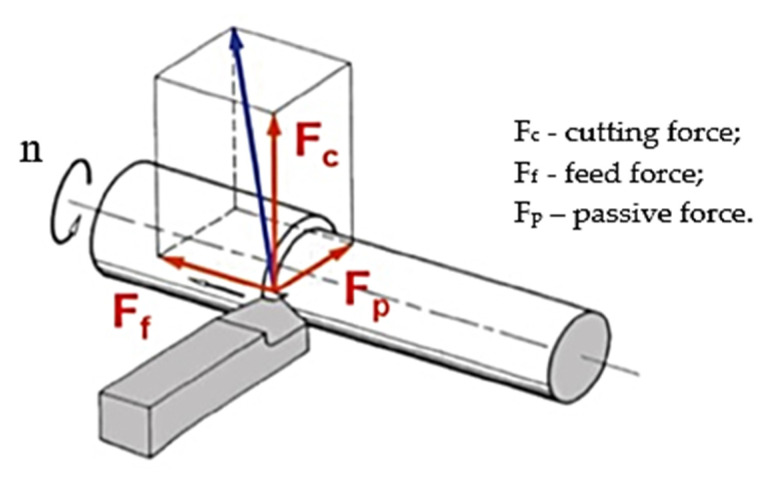
The components of the cutting force at longitudinal turning.

The cutting forces appear as a result of the elastic and plastic deformation of the splinter and the machined surface, for breaking, detaching, additional deformation (bending and spiraling) of the splinter as well as overcoming the frictional forces between the splinter and the clearance face and between the face of the settlement and processed surface. How the components of the cutting force act on the longitudinal turning are shown in Figure 3.

Cutting forces are a limiting factor of machinability, they influence the energy consumption during the processing of parts. High cutting forces can cause temporary deformations of the tool and the piece and can cause vibrations and permanent deformations of the blank (loss of machining tolerances). The research aimed to establish the way in which the cutting forces evolve depending on the variation of the functional geometry of the cutting tool during the turning operation. For the mathematical modeling of the evolution of the force magnitude according to the geometry of the cutting tool, it was started from the equation [30]:

(1) $$F_{i}=K_{i11}\cdot \;a_{p}\cdot f^{i-c}\left(\sin\chi_{r}\right)$$

where: *K_i_*_11_ is the specific cutting force; $\chi_{r}$ cutting edge angle; *i*–*c* exponent that is determined experimentally.

To simplify Equation (1) and given the significance of the coefficients and exponents, the following notation was made:

(2) $$K_{i11}\cdot \;a_{p}\cdot f^{i-c}=k$$

Thus Equation (1) becomes:

(3) $$F_{i}=k\;\cdot \;\sin\chi_{r}$$

The angle of the main cutting direction *η* can be expressed with a relation of shape [30]:

(4) $$\eta =arctg(\frac{f}{\pi D_{M}}\cdot \sin\chi_{r})$$

where: *D_M_* is the diameter of an M point on the surface of the workpiece

Given Equations (3) and (4), respectively, Equation (5) was obtained, which shows the dependence of the cutting force on the angle of the main cutting direction *η* and the diameter of a point on the workpiece *D_M_*.

(5) $$F_{i}=k\cdot \frac{tg\eta \cdot \pi \cdot D_{M}}{f}$$

Modeling of cutting forces is essential to predict the progress of machining operations as well as the final properties of the workpieces. On a large scale, cutting forces can be used to size the clamping system or to ensure the geometry and roughness of the machined surface. 

Measurement of forces and modification of numerical control (NC) instructions may be used in some cases, but other methods are preferred due to the cost of monitoring equipment and difficulties in modifying control data. Consequently, new methods of controlling cutting forces that can be used for various cutting operations are needed.

**Figure 4:**
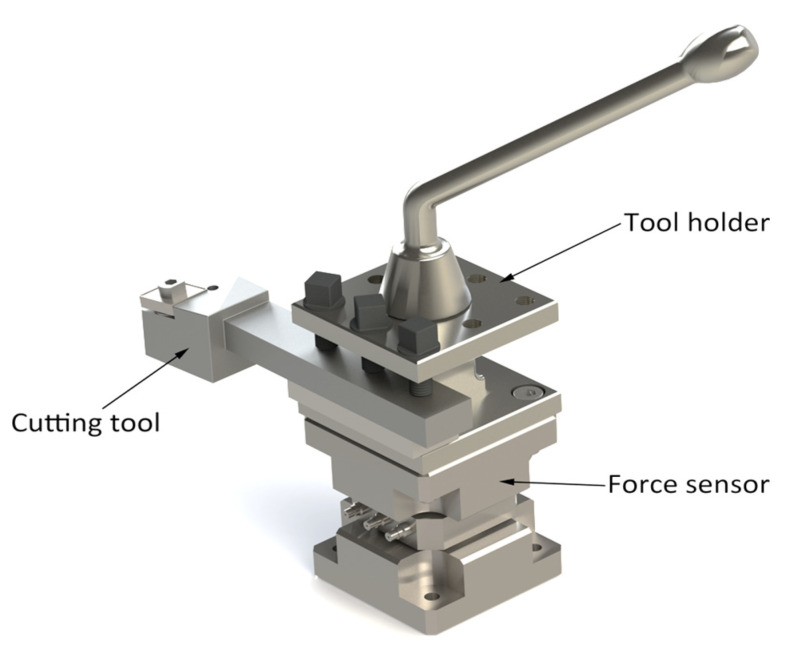
The system used to measure cutting forces.

Thus, with the help of Equation (5) it is possible to establish a range of variation of the values of the functional geometry of the tool so that the cutting force has values as small as possible and the amount of energy consumed in cutting is as small as possible. During the research, for the monitoring of the cutting forces, the system presented in Figure 4 was used, which allows the determination of the cutting forces in the 3 directions. A piezocapacitive force sensor, PCB 261A13 from PCB, was used to measure the forces, which allows measurement in both dynamic and quasi-static mode. The sensor has a capacity of 70 pF. In the Z direction, it allows the measurement of a maximum force of 44.48 kN and in the X and Y directions a maximum force of 19.57 kN. Prior to measuring the forces in the turning process, the sensor was calibrated by measuring static forces in the range of values of the forces measured in the process. A traction-compression test machine Instron 5587 was used for calibration. The electrical signal transmitted by the force sensor is taken by means of a low-noise cable to the digital charge amplifier CMD 600, produced by HBM. The amplified signal is transmitted to the Quantum X MX840B acquisition system also from HBM. The Catman software package of the acquisition system was used to acquire, process and measure the forces.

After establishing the values of the forces, it was possible to calculate the power required for cutting. Power required for cutting *P* refers to the power consumed when removing material in the form of chips to which is added the additional loss of power of the machine tool. Given [24], the calculation of power can be carried out with the following equation:

*P* = *P_cut_* + *P_ad_* (6)

where: *P_cut_* is the power required to remove the material; *P_ad_* = *α·P_cut_*—the load loss; α- The additional power loss coefficient that can be considered that have a fixed value for a CNC machine tool. Thus, the power calculation relationship becomes:

*P* = *P_cut_* ·(1 + *α*) (7)

According to [30], the calculation relation of the power consumed for the removal of the material to be processed is:

(8) $$P=\frac{F_{c\cdot v_{c}}}{6000}$$

where: *F_c_* is the cutting force [daN}, *v_c_* is the cutting speed [m/min].

In order to observe the energy consumption in the baldness performed, only *P_cut_* was considered, considering that the value of the coefficient α can be considered constant. 

### 2.4. Measurement of the Roughness of the Processed Surfaces

The roughness of the surfaces machined depends on many parameters of the machining process, but, among them, in the processing of bronze and aluminum, the phenomenon of deposits on the edge is very important, respectively, the friction phenomena. Thus, the roughness is determined mainly by glossy and hard deposition scales, representing particles from the deposits on the edge embedded in the generated surface, respectively, craters or porosities corresponding to the areas from which hard particles embedded in the base mass of the part were extracted. Furthermore, the geometry of the tool influences the size of the roughness, and in the research two geometric parameters were taken into account, namely: the clearance angle and the seating angle. Laying angle α influences the roughness by the plastic deformations of the machined surface as a result of the contact surface with respect to the placement/machined surface. By increasing the angle *α*, the radius *r_n_* of the edge decreases, the contact surface will be smaller and as a result the plastic deformations will decrease and the roughness *R_a_* will decrease. The clearance angle γ, influences the roughness of the processed surface by means of plastic deformations, including by the phenomenon of deposits on the edge. Thus, the area of the tool tip influences the intensity of the plastic deformations and directly contributes to the formation of the roughness of the machined surface. From a mathematical point of view, the dependence of the roughness on the constructive geometry and the functional geometry of the tool can be expressed with the relations [21]:

(9) $$R_{a}=\frac{C_{v}\cdot K_{v}}{T^{xv}\cdot v_{f}}\cdot tg(\gamma_{Fe}-\gamma )$$

(10) $$R_{a}=\frac{C_{v}\cdot K_{v}}{T^{xv}\cdot v_{f}}\cdot tg(-\alpha_{Fe}+\alpha )$$

where: *C_v_*; *K_v_*; *x_v_* is the coefficients; *T*—tool durability; *V_f_*—feed rate; *γ*—rake angle, *α*—clearance angle; *α_Fe_*—effective clearance angle; *γ_Fe_*—effective rake angle.

The constructive modification made to the tools was aimed at ensuring both an optimal functional geometry, which would avoid the occurrence of the phenomena of deposits on the edge, and the decrease of surface roughness and frictional forces, with effects on reducing energy consumption.

An SJ-500P system produced by Mitutoyou was used to measure roughness. This measuring system is extremely versatile and the software offers high accuracy and performance. 

**Figure 5:**
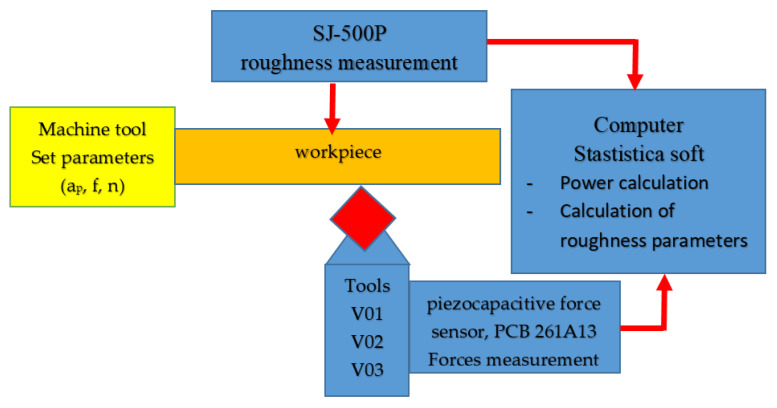
Experimental Setup.

In view of the above, a necessary system was designed and used in the experimental research, Figure 5. Thus, the system shows how the parameters of the cutting regime are regulated, the equipment used to measure forces and roughness, but also computing equipment and software used to process experimental data.

## 3. Results and Discussion

The experimental research aimed at establishing the value of the forces and the roughness of the surfaces, taking into account the establishment of some correlations between them. In addition, for a confirmation of the results obtained in the experimental research, 10 samples were processed in the same conditions, taking into account the three variants of cutting tools (V01; V02; V03). The research mainly pursued reducing energy consumption while ensuring good surface quality. Thus, in the first stage of the research, a monitoring of the values of the cutting forces was performed according to the three directions, taking into account the three tool variants. Following the monitoring of the force values, a measurement of the roughness of the processed surfaces was made so that a correlation between them could be observed. In order to be able to make a more complete analysis of the correlation between the forces and the roughness, the experimental data were processed with the help of the STATISTICA software.

### 3.1. Analysis of the Values of the Forces That Appear during the Cutting Process

The measurement of the values of the cutting forces was performed in the three directions shown in Figure 4. Thus, 10 samples of the same type were processed for each variant of cutting tool and cutting regime, according to those presented in Table 3. The values of the measured forces were established as an average of the 10 values for each of the 8 samples. The use of average force values allows an improvement in the adequacy of the experimental results. Furthermore, the experimental results obtained can highlight the effect that the constructive solutions of cutting tools have on the size of the cutting forces. Each sample was also processed to a length of 25 mm. Modification of values of the cutting forces, especially, is determined by ensuring an optimal geometry of the cutting tool throughout the cutting process. 

The analysis of the values of the cutting forces is a very good method of evaluating the phenomena that accompany the cutting process. Thus, their values provide information on the degree of stress of the cutting edge of the tool, but also of the frictional forces that accompany any manufacturing process by cutting.

The medium values obtained for the three components of the cutting forces for the 3 variants of cutting tools are presented in Table 4 for *F_c_*, Table 5 for *F_f_* and Table 6 for *F_p_*.

The results of the experimental research have shown that the use of tool construction variants can lead to a reduction in the value of the cutting forces with effects and on the reduction of energy consumption. Thus, the largest reductions in the value of the cutting forces were obtained if the constructive tool version V03 was used. Small reductions in the values of the cutting forces were also obtained in the case of the V02 variant, and this can be explained by the fact that the use of only the spring washer does not ensure an optimal tool geometry during cutting. In case of using the tool in variant V03, an optimal tool geometry can be achieved due to the fact that the plate can self-regulate its position with 3°. This possibility of adjusting the position of the plate results in a substantial reduction in the frictional forces on the clearance face or the main seating face. Thus, from the obtained results it was observed that the substantial reduction of forces was achieved for component *F_c_* and *F_f_*, respectively, and in the case of component *F_p_* the reduction of its value was very small. 

Using the tool V03 makes the actual tool angles for each edge segment optimal. All this confirms that the optimal effective tool angles also allow an optimal distribution of the force intensity. In addition, a more uniform distribution of force along the edge allows a better repartition of heat with positive effects in the sense of reducing deposits on the edge [31]. Thus, the use of the V03 variant reduces the tendency of this material to form deposits on the edge and, at the same time, there is a tendency to increase the forces due to the increase of the radius of the tool tip thus confirming the results obtained by other researchers [32]. It should be noted that the presence of deposits on the edge also causes an increase in the contact area between the workpiece and the tool, with effects on the increase of the frictional forces, respectively, of the cutting forces.

By reducing the values of the F_c_ component, a reduction in energy consumption can be obtained due to the fact that this is one of the most important parameters that influence the size of the power consumed in cutting. The presence of very high frictional forces also determines the release of high frequency energies in the cutting area, with negative effects on the roughness of the parts’ surfaces. Thus, the use of the V03 tool, which allows a reduction in the size of the forces by about 30%, creates conditions to reduce the temperature of the elements of the technological system, but also a decrease in the amount of high frequency energy with positive effects on the roughness of processed surfaces.

The measured values for the cutting forces show that, indeed, they are directly influenced by the angle values of the main cutting direction η according to relation 5. Thus, the V03 cutting tool variant allows the adjustment of an optimal value for the angle η, which allows to obtain the lowest value for the cutting forces. 

After obtaining the values of the forces, a calculation of the power consumed at cutting was made, taking into account the relation 8. By establishing the value of the power consumed at cutting, the effects of the adoption of a certain design for tool on energy consumptions can be better observed. The values obtained for the average power required for cutting considering the 3 tool variants are presented in Table 7.

The results presented in Table 7, regarding the cutting power, were statistically processed using the Statistics software. Thus, a Multiple Regression analysis was performed. The parameters F and *p* have been established, respectively, and the results obtained are presented in Table 8.

From the analysis of the results presented in Table 7 it is observed that the largest reduction, of approximately 30%, of the value of the cutting power was obtained in the case of the S5 sample, processed with tool V03. It should be noted that the largest reduction was obtained when the highest cutting power values were also calculated. This is all the more important because the maximum yield was obtained for the sample processed with cutting regimes specific to roughing operations. Given that, in addition to the reduction in cutting power, there is also an improvement in the quality of the machined surfaces, the advantage of using the V03 tool in machining is demonstrated. 

With the help of the parameters established following the Multiple Regression analysis F and *p*, a series of conclusions can be summarized regarding the way in which the parameters of the cutting regime influence the values of the cutting power. Thus, higher values of F and lower values for *p* (*p* < 0.1) indicate that the corresponding variable is very significant [33,34]. From the values presented in Table 8 it is observed that the cutting depth has the greater influence on cutting power. It is also noted that, for tool V03, the value of *p* is the lowest and the value of F is the highest. In these circumstances, it can be concluded that the V03 tool can be used with the best results in the case of roughing processes.

### 3.2. The Influence of the Use of Smart Tools on the Roughness of Surfaces Machined through Cutting

The results of previous research have shown that the roughness of the surfaces is influenced by both the feed rate and the cutting speed and the cutting depth. Thus, considering the calculation relationship of the cutting force that depends on the three factors mentioned above, it can be concluded that the cutting force together with the friction forces have a very large influence on the surface roughness [35]. In addition, the surface roughness of a part made of aluminum bronze materials can depend in particular on the combination of the material of the workpiece and of the tool, the geometry of the tool and the vibrations that may occur in the cutting process [36].

Cutting forces and surface roughness are two very important aspects that must be taken into account when processing aluminum bronze materials. Thus, cutting forces have an important influence on the specific cutting pressure and energy consumption [37]. Machining involves a large number of process variables, but in particular, it is always necessary to optimize the cutting force and surface roughness in order to turn the process into an eco-process.

Under these conditions, research has sought to improve conditions by ensuring minimum values of shear strength and surface roughness. This was mainly due to the fact that the ecodesign of a cutting process differs according to each type of material processed, and the analyzed material has been very little studied in previous research. Thus, the optimization of the aluminum bronze cutting process was carried out to establish a correlation between the cutting force and the surface roughness under the conditions of a minimum energy consumption. Very often there is a connection between the cutting forces, the friction forces, the phenomenon of deposits on the edge and the roughness profile. The adoption of constructive solutions for the cutting tool can allow the creation of conditions that avoid the phenomenon of deposits on the edge. Thus, the research aimed to achieve a tool modified constructively compared to the classic version, which would obtain the best roughness in terms of minimum energy consumption and aimed to create a smart tool to control the phenomenon of deposits on the edge.

**Figure 6:**
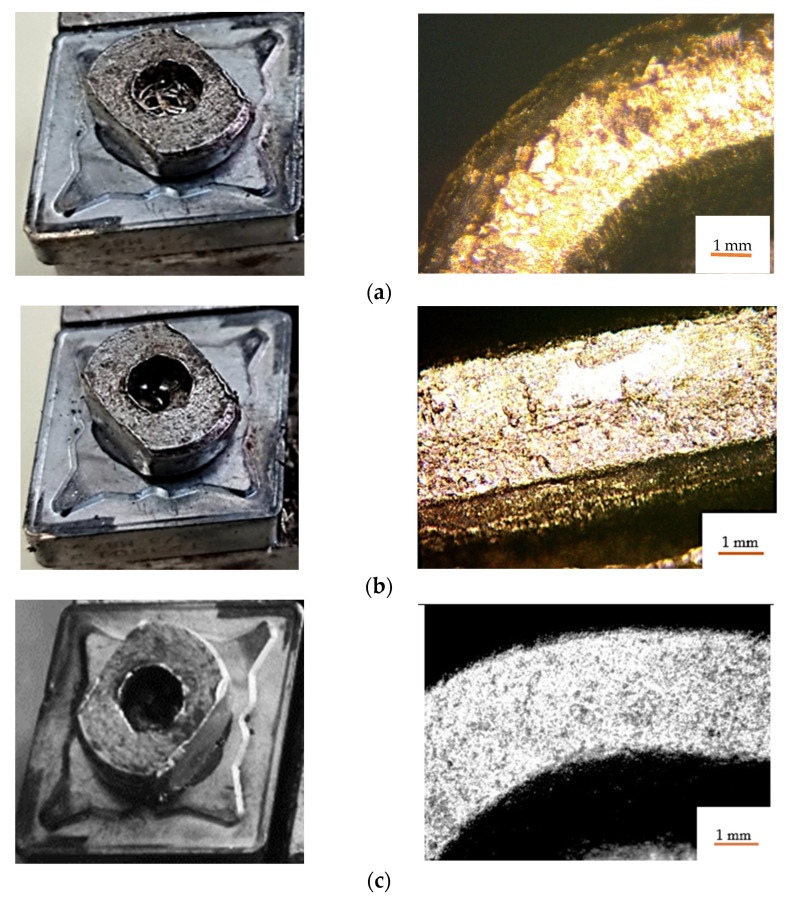
Deposits of processed material on the tool edge: (**a**)—for tool V01; (**b**)—for tool V02; (**c**)—for the tool V03.

The results obtained showed that, if the V01 tool was used, the deposition of the processed material on the tool edge was quite pronounced, Figure 6a; if tool V02 was used the deposition of processed material on the tool edge was insignificant, Figure 6b; if the V03 tool was used, no deposits appeared on the tool edge, Figure 6c.

The analysis of the surface roughness was performed for the same processing parameters considering the three constructive variants of tools presented in Figure 1. This analysis was necessary because the constructive changes brought to the tools can substantially influence both the size of the cutting forces, respectively, friction, and the size of the roughness of the machined surfaces. Under these conditions, a change in the design of the tool can lead to the transformation of the cutting process into an eco-process. 

In order to be able to establish a correlation between the cutting forces and the surface roughness, the roughness measurement was performed for the same samples for which the cutting forces were also measured. The determination of the roughness was made by repeating the measurements for the 10 samples made with each type of tool. In order for the roughness measurement to be as accurate as possible, the roughness was measured on two diametrically opposed surfaces, and this was carried out by indexing the samples with 360°. As in any research activity, there is a possibility that certain values may be aberrant and thus an analysis has been performed to eliminate them, as they may lead to disproportionate results from statistical processing. The Minitab program was used for statistical data processing, and aberrant results were eliminated with the help of the boxplot analysis. In addition, using the same statistical processing program, the normality of the data distribution was verified using the calculation of the mean, the median value and the standard deviation. The Kolmogorov-Smirnov test and the Andreson-Darling test were used to verify the normality of the experimental results. The choice of their use for the verification of normality was made considering the fact that they are the most sensitive tests used for the verification of the normal distribution.

**Figure 7:**
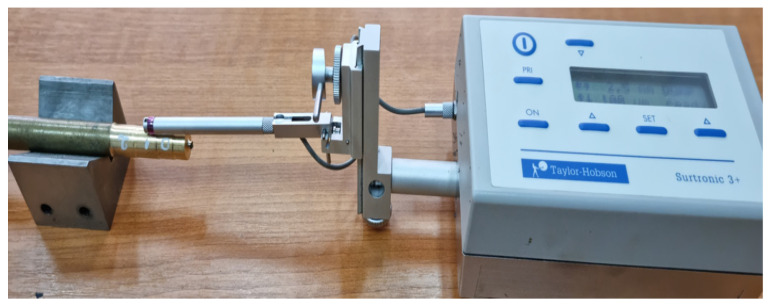
Images with surface roughness obtained by cutting machining.

The surface roughness was measured for 10 samples processed under the same conditions, and this was required to ensure the adequacy of the results obtained. Each of the 10 specimens was processed with the 3 constructive variants of tools (V01, V02, V03). The roughness values measured for the 5 sample processed with the 3 types of tools are shown in Table 9. The choice of roughness measurement for sample 5 was made given that the largest reduction in measured shear strength was observed in this case. Thus, it was observed whether tool V03 allows, in addition to a reduction in energy consumption and an improvement in the quality of the processed surfaces. The roughness measurement process of the parts made of bronze aluminum was performed according to those shown in Figure 7, and the sample presented during the roughness measurement was number 5, for which the lowest value for Fc and the lowest roughness of the processed surface were obtained. 

**Figure 8:**
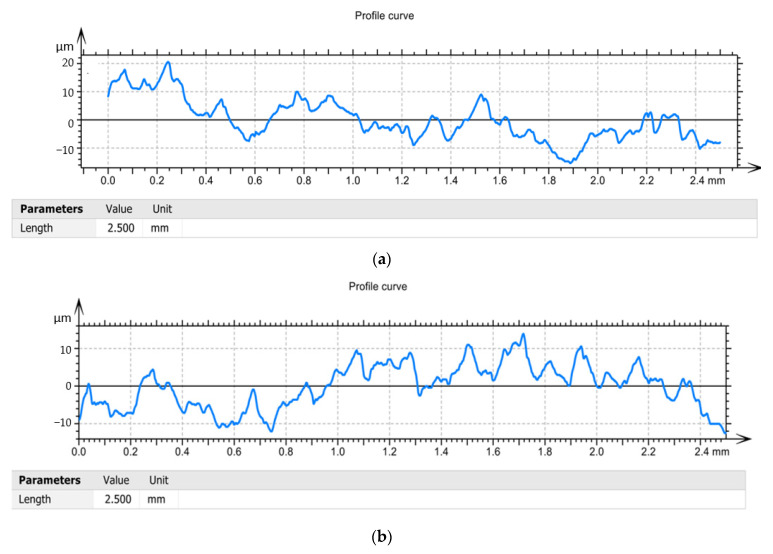

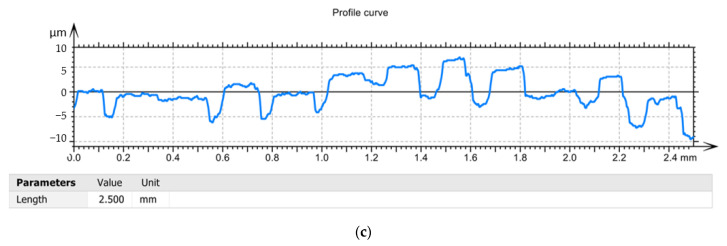
Profile curve: (**a**)—in the case of machining with a V01 cutting tool; (**b**)—in case of machining with V02 cutting tool; (**c**)—in case of machining with cutting tool V03.

According to relations (9) and (10), respectively, the roughness of the machined surface depends very much on the speed of the parameters of the cutting regime and on the geometry of the cutting tool. Regarding the geometric parameters, it is very important to always keep optimal values for the setting angle (α) and clearance angle (γ) [38,39,40]. Thus, by adopting improved tool construction variants (V02, V03), conditions are created for maintaining the best possible functional geometry of the tool during machining. So, both the V02 variant and the V03 variant allow the modification of the values of the setting angle (α) and the clearance angle (γ) within certain limits, but the tool in the V03 variant has the best conditions for adjusting the geometry. The values presented in Table 9 demonstrate that the tool V03 allows the lowest roughness to be obtained. This is explained by the fact that the arrangement of the plate with a support whose axis can change its position by 3° allows to ensure an optimal functional geometry and, at the same time, determines a reduction of the frictional forces. It should be noted that the reduction in roughness is considerable, by about 80–90% when using tool V03 compared to tool V01. It is also noted that, if the tool V02 was used in the processing, the reduction in roughness is insignificant, which can be explained by the fact that in the case of this tool can not ensure optimal geometry but only some damping of vibration. Given the values of the roughness and the measured forces, it was found that there is a correlation between the roughness and the cutting force in the sense that, for the case when the V03 tool was used, the best roughness and the largest reduction in cutting power. From the analysis of the Profile curve diagrams, Figure 8 it was observed that the maximum value of the roughness was obtained in the case of the tool V01 of 20 µm, and the lowest value in the case of the use of the tool V03 of 5 µm. This can be explained by the fact that the cutting process in this case is a dynamic one, mainly determined by formation and breaking at certain time intervals of the deposits on the edge. In the case of using tools V02 and V03, the roughness values are more stable along the processed surface, Figure 8b,c, respectively, and this can be explained by the fact that the dynamic phenomena caused by the appearance of deposits on the edge are eliminated to a large extent. However, the roughness values are lower in the case of the V03 variant because in the case of this tool conditions are created to ensure an optimal tool geometry during cutting.

**Figure 9:**
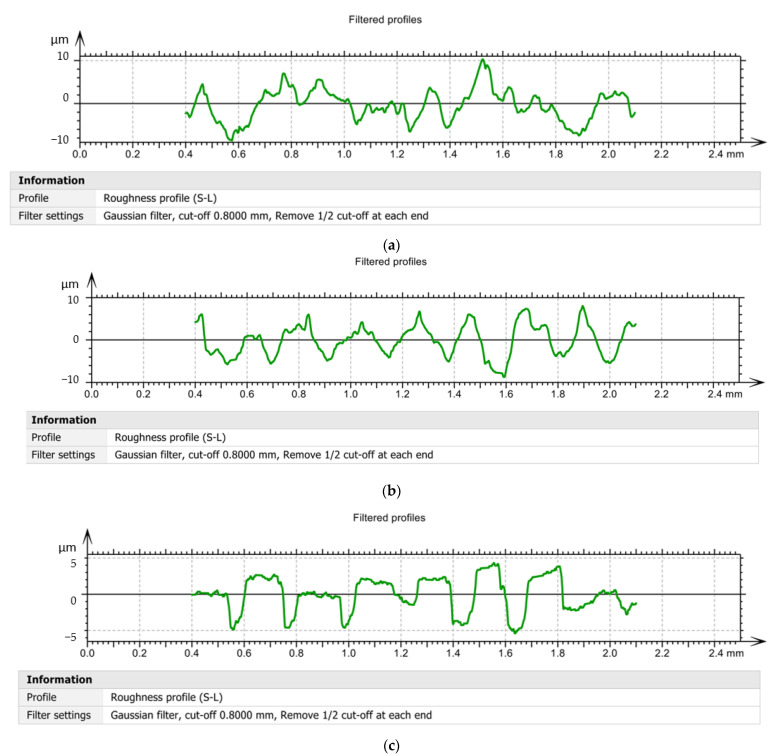
Filtered profiles: (**a**)—in the case of machining with a V01 cutting tool; (**b**)—in case of machining with V02 cutting tool; (**c**)—in case of machining with cutting tool V03.

The experimental data obtained during the roughness analyzes were processed and a series of curves related to the filtered profiles were obtained, Figure 9. During the research, it was decided to draw the filtered profile curves, because they are obtained by eliminating those wavelengths located outside the band of interest. Thus, filtered profiles obtained when using the tool V01, Figure 9a, demonstrate that the roughness values have very large variations in the length of the surface and reach values of ±10 µm. This demonstrates that the choice of a constructive variant of tool V01, determined both a high roughness and a large variation of it along the measured surface. If the tool V02, Figure 9b, respectively, the tool V03 was used, it was possible to obtain filtered profiles that were much more stable and with a much smaller amplitude than the situation in which the tool V01 was used.

It should be noted that the results obtained in the case of filtered profile tracing are consistent with those obtained in the case of curved profile tracing, and this shows that the elimination of wavelengths outside the band of interest does not lead to a substantial change in the results obtained. Furthermore, the comparison of the values obtained for the roughness with those obtained for the cutting forces confirms the direct connection between the forces that appear during the processing process and the roughness of the surfaces [41,42].

**Figure 10:**
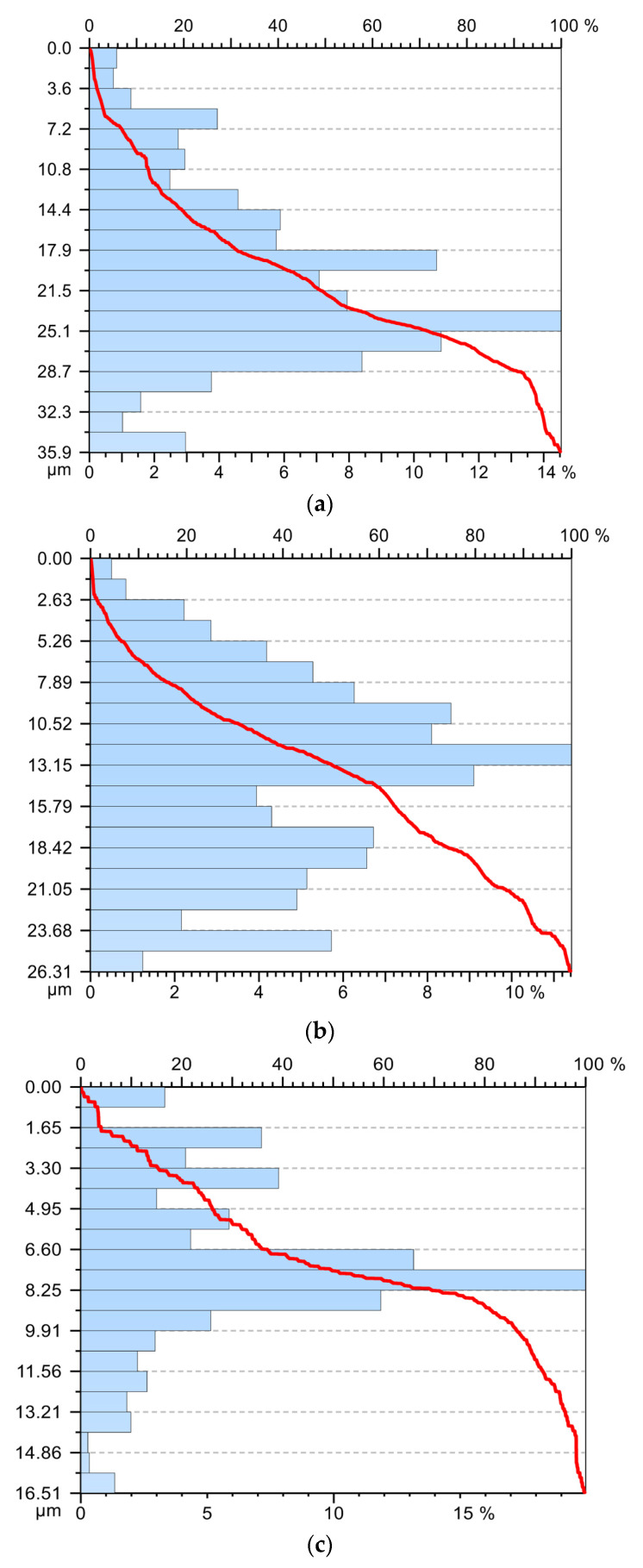
Abbott Firestone curve: (**a**)—in the case of machining with a V01 cutting tool; (**b**)—in case of machining with V02 cutting tool; (**c**)—in case of machining with cutting tool V03.

For the 5 sample, the experimental data obtained were processed and the graphs, Abbott Firestone curve, presented in Figure 10. The analysis of the Abbot Firestone curve demonstrates that, in the case of tool V01, Figure 10a, the best stability of the values of roughness is not obtained, and their distribution does not fall within a normal distribution. If the tools V02 and V03 were used, respectively, a fairly good distribution of roughness values was obtained, coming quite close to the normal distribution, especially if the tool V03 was used, Figure 10c. All this demonstrates that the use of the V03 tool provides the best stability for the cutting process with positive effects on surface roughness and cutting forces. Thus, they allow the transformation of the cutting process into an eco-process.

Experimental research shows that, in the conditions in which certain parameters of the cutting process are optimized, it can be transformed from a processing process into an eco-process. This is possible by the fact that, in addition to a reduction in surface roughness, a reduction in cutting forces and, consequently, in the amount of energy consumed in cutting can also be achieved. It should be noted that, although only the constructive form of the tool has been optimized and yet the results obtained have been promising, further optimizations can be made regarding other parameters accompanying the machining processes (parameters of the cutting regime, the presence of lubricating coolants, tool material, etc.).

It should be noted that the use of the V03 tool allows to obtain the lowest power consumed in cutting but also a very large difference in roughness. Thus, sample S5 has been processed with a cutting regime specific to the roughing operation, but the results obtained for roughness are remarkable. Under these conditions, the processing of bronze-type parts with the V03 tool can be carried out in a single roughing phase to ensure the quality of the machined surfaces. Although the use of a low cutting speed can lead to the appearance of sharp deposits when using the V03 tool, this phenomenon has not occurred. This can be explained by the fact that the use of the V03 tool can provide an optimal value for the clearance angle that has the greatest influence on the deposition on the edge. The lack of deposition on the edge has a positive influence on the surface roughness because there are no longer certain detachments of the material from the deposition on the edge and its adhesion on the processed surface. A predicted square error (PSE) criterion was proposed to correlate the shear force with the surface roughness in other research [43]. This criterion could also be used in the research presented as it would help us to determine the surface roughness and shear force using a prediction model. In view of the results obtained, future research could identify a new criterion for establishing predictions for surface roughness and shear forces. In addition, some optimization methods can be used, such as the Taguchi method, which can be used successfully to identify the optimal cutting parameters and the optimal tool geometry that obtain the lowest roughness [44,45].

Under these conditions, research has shown that there is a possibility that, by optimizing the constructive form of the tool, a processing process can be transformed into an eco-process. 

## 4. Conclusions

The research aimed at designing a tool for machining materials such as bronze-aluminum alloys to reduce energy consumption while ensuring a very good quality of the processed surfaces.

Thus, it has been shown that it is possible to reduce energy consumption and turn the processing into an eco-process. In addition, the steps taken in the research allowed to establish the optimal conditions for which the lowest energy consumption and the lowest roughness of the processed surfaces can be obtained. The ecodesign of the cutting process of the bronze-aluminum alloy cutting has demonstrated the following:

- the maximum reduction in cutting forces was about 30%, and this reduction also allows a decrease in cutting power and, implicitly, in the amount of energy consumed;
- the effect of the constructive changes brought to the cutting tool also determines a reduction of the intensity of the adhesion phenomenon of the material to be processed on the cutting edge of the tool;
- by reducing the adhesion of the processed material on the cutting edge of the tool, an improvement of the surface roughness of the part was also obtained, thus achieving a correlation between energy consumption and surface roughness,
- there is the possibility to choose the design parameters that allow the transformation of the processing process into an eco-process.
- tool V03 allows to obtain the best performances if it is used in the roughing processes.

The research presented has shown the importance of adopting the principles of eco-design for the process of cutting aluminum alloys by cutting, but these results can also be used in eco-design of processing processes for other types of alloys. Future research will aim to analyze the possibilities of applying the results obtained for other types of tools and processing procedures.

## Figures and Tables

**Table 1:** The chemical composition and mechanical properties of aluminum bronze (C62300).

Density Aprox. kg/dm^3^	Composition, %	Tensile Strength, N/mm^2^	Yeld Stress, N/mm^2^	Elongation, %	Brinell Hardness
7.6	Al = 9.5–10.7	>578	cca. 325	>12.5	168–172
	Fe = 2.1–3.8				
	Mn 1.6–3.4				
	Cu–balance				

**Table 2:** Tool parameters of SNMG 12 04 12-PMC—Sandvik Coromant.

Plate Type	Rake Angle	Clearance Angle	Cutting Edge Angle	Minor Edge Angle
SNMG 12 04 12-PMC	6°	8°	85°	5°

**Table 3:** Programming experiments through the method of factorial experiments.

Sample Number	*n* [rpm]		*a_p_* [mm]		*f* [mm/rot]	
	800	1200	0.4	0.8	0.15	0.25
S1	x			x	x	
S2		x	x			x
S3		x		x	x	
S4		x		x		x
S5	x			x		x
S6	x		x			x
S7		x	x		x	
S8	x		x		x	

**Table 4:** The medium values of the *F_c_* component for the 3 variants of cutting tools, daN.

No. of the Sample	The Constructive Variant of the Tool		
	V01	V02	V03
S1	86.21	68.91	59.54
S2	62.74	57.16	44.90
S3	83.17	71.97	61.36
S4	123.44	97.63	89.81
S5	119.18	93.11	87.32
S6	70.19	55.83	47.83
S7	44.79	39.77	30.79
S8	35.48	33.16	27.51

**Table 5:** The medium values of *F_f_* component for the 3 variants of cutting tools, daN.

No. of the Sample	The Constructive Variant of the Tool		
	V01	V02	V03
S1	50.13	41.95	25.29
S2	37.42	35.29	27.56
S3	49.12	41.07	25.35
S4	74.96	58.31	52.11
S5	70.51	57.72	49.32
S6	43.86	36.93	28.18
S7	28.39	22.01	18.17
S8	21.68	18.07	16.29

**Table 6:** The medium values of *F_p_* component for the 3 variants of cutting tools, daN.

No. of the Sample	The Constructive Variant of the Tool		
	V01	V02	V03
S1	34.53	68.91	59.54
S2	25.98	57.16	44.90
S3	33.17	71.97	61.36
S4	53.41	97.63	89.81
S5	48.29	93.11	87.32
S6	29.56	55.83	47.83
S7	26.63	39.77	30.79
S8	21.11	37.16	33.51

**Table 7:** The medium values of the power, *P*, for the 3 variants of cutting tools, kW.

No. of the Sample	The Constructive Variant of the Tool		
	V01	V02	V03
S1	1.081	0.865	0.714
S2	0.525	0.478	0.375
S3	0.696	0.602	0.513
S4	1.033	0.817	0.751
S5	1.504	1.169	1.096
S6	0.881	0.701	0.573
S7	0.374	0.332	0.257
S8	0.445	0.416	0.345

**Table 8:** Parameters F and *p*, respectively, after analysis Multiple Regression.

Parameters of the Cutting Regime	V01		V02		V03	
	F	*p*	F	*p*	F	*p*
n	1.513	0.253	1.491	0.265	1.151	0.302
a_p_	6.752	0.041	6.753	0.035	8.597	0.019
f	1.611	0.339	1.621	0.305	1.731	0.585

**Table 9:** The measured roughness values for the S5 sample, Ra (µm).

No. of the Sample	The Constructive Variant of the Tool		
	V01	V02	V03
1	3.242	2.823	1.647
2	2.975	2.984	1.677
3	2.853	3.050	1.685
4	3.081	3.235	1.581
5	2.780	3.150	1.877
6	3.267	3.047	1.904
7	2.997	2.963	1.650
8	3.201	2.814	1.401
9	3.260	2.929	1.894
10	3.023	3.086	1.504
Mean	3.068	3.008	1.682
StDev	0.172	0.134	0.168
Cvariation	5.635	4.455	3.012
Median	3.052	3.015	1.664
*p*-value	0.449	0.911	0.36

## Data Availability

Not applicable.
